# Construction and Verification of a Risk Prediction Model for the Occurrence of Delayed Cerebral Ischemia after Aneurysmal Subarachnoid Hemorrhage Requiring Mechanical Ventilation

**DOI:** 10.1155/2023/7656069

**Published:** 2023-02-17

**Authors:** Xianjun Chen, Yue Ou, Jingxing Leng, Changfeng Wang, Feixiang Min, Yinghua Xia, Gui Yu, Hui Xiang, Ru'en Liu

**Affiliations:** ^1^Medical College of Nanchang University, Nanchang, Jiangxi 330006, China; ^2^Department of Neurosurgery, Jiangxi Provincial People's Hospital, Nanchang, Jiangxi 330006, China; ^3^Jiangxi Provincial People's Hospital, The First Affiliated Hospital of Nanchang Medical College, Jiangxi 330006, China; ^4^The First Clinical Medical College, Gannan Medical University, Ganzhou, Jiangxi 341000, China; ^5^Department of Neurosurgery, Peking University People's Hospital, 11th Xizhimen South St., Beijing 100044, China

## Abstract

**Objectives:**

Delayed cerebral ischemia (DCI) contributes to poor aneurysm prognosis. Subarachnoid hemorrhage and DCI have irreversible and severe consequences once they occur; therefore, early prediction and prevention are important. We investigated the risk factors for postoperative complications of DCI in patients with aneurysmal subarachnoid hemorrhage (aSAH) requiring mechanical ventilation in intensive care and validated a prediction model.

**Methods:**

We retrospectively analyzed patients with aSAH who were treated in a French university hospital neuro-ICU between January 2010 and December 2015. The patients were randomized into a training group (144) and verification groups (60). Nomograms were validated in the training and verification groups, where receiver operating characteristic curve analysis was used to verify model discrimination; calibration curve and Hosmer-Lemeshow test were used to determine model calibration; and decision curve analysis (DCA) was used to verify clinical validity of the model.

**Results:**

External ventricular drain (EVD), duration of mechanical ventilation, and treatment were significantly associated in the univariate analysis; EVD and rebleeding were significantly associated with the occurrence of DCI after aSAH. Binary logistic regression was used to select five clinicopathological characteristics to predict the occurrence of DCI in patients with aSAH requiring mechanical ventilation nomograms of the risk of DCI. Area under the curve values for the training and verification groups were 0.768 and 0.246, with Brier scores of 0.166 and 0.163, respectively. Hosmer-Lemeshow calibration test values for the training and verification groups were *x*^2^ = 3.824 (*P* = 0.923) and *x*^2^ = 10.868 (*P* = 0.285), respectively. Calibration curves showed good agreement. DCA indicated that the training and verification groups showed large positive returns in the broad risk range of 0-77% and 0-63%, respectively.

**Conclusions:**

The predictive model of concurrent DCI in aSAH has theoretical and practical values and can provide individualized treatment options for patients with aSAH who require mechanical ventilation.

## 1. Introduction

Aneurysmal subarachnoid hemorrhage (aSAH) is a dangerous and devastating cerebrovascular event with a mortality rate of up to 50% [1]. Patients diagnosed with aSAH are treated mainly for intracranial aneurysms with cranial clamping or interventional surgical vascular embolization [2, 3]. However, despite timely surgical treatment, the vast majority of surviving patients require treatment, such as mechanical ventilation in intensive care, and a variety of postoperative complications can still occur, with delayed cerebral ischemia (DCI) being a common postoperative complication [4, 5].

Delayed cerebral ischemia, as one of the main causes of neurological impairment after aneurysmal subarachnoid hemorrhage, was once understood to be a sequela caused by cerebral vasospasm alone, and some scholars have even defined DCI directly as a poor prognosis for the occurrence of cerebral vasospasm [6, 7]. However, current studies have shown that the pathogenesis associated with DCI is much more than that, and the mechanisms that have gained more recognition, including early brain injury, cortical diffusion depolarization, microthrombosis, microcirculatory spasm, impaired cerebral blood flow autoregulation, oxidative stress, and reactive oxygen radical generation, and other mechanisms (such as cell death, inflammatory response, and blood-brain barrier disruption) may all contribute to the development of DCI [8–11].

Improvements in available neurocritical care equipment and techniques have led to a slight decrease in the overall prevalence and mortality of aSAH in recent years [12]. Reversing DCI as soon as possible before the ischemic process progresses to cerebral infarction requires clinicians to detect DCI early, which is a critical step for effective intervention [13]. Therefore, an early and accurate prediction of DCI and poor prognosis is crucial for the timely intervention of patients.

Nomograms are reliable statistical models that construct graphical predictive tools that incorporate several risk factors for the development of the disease and provide personalized, easily accessible, evidence-based, and highly accurate risk assessments for targeted clinical decision making [14]. Even in many cancers, the predictive accuracy of nomograms is higher than that of various traditional staging systems [15–17].

Patients with aneurysmal subarachnoid hemorrhage are critical and require intensive care, and mechanical ventilation is a routine treatment in intensive care [18, 19]. The assessment and risk factors for the development of DCI after surgery in patients with aSAH who require mechanical ventilation have not been studied, and there is no specific risk screening tool for this group of patients. This study developed and validated a risk prediction model that facilitates the use of simple scoring and calculation methods to predict the likelihood of DCI after aSAH based on variables of interest during the patient's hospitalization regarding reporting specifications for the development, validation, and updating of clinical prediction models and explored its predictive application for the development of DCI in patients with aSAH who require mechanical ventilation to provide a theoretical basis for reducing the risk incidence.

Our research is a post hoc analysis of a dataset shared by Chalard et al. [20]. Under the terms of service of the Dryad repository (https://datadryad.org), researchers can reasonably apply the data according to different research hypotheses.

We will analytically review the impact of age, sex, location of the responsible aneurysm (anterior/posterior circulation), surgical approach, time to diagnosis and treatment, rebleeding, external ventricular drain (EVD), angiographic vasospasm, duration of mechanical ventilation, and admission and follow-up-related rating scales on the incidence of DCI after aneurysmal subarachnoid hemorrhage in patients.

The DCI-related nomograms were constructed based on the identification of relevant risk factors, and the feasibility of the model was validated by three dimensions: discrimination, calibration, and clinical decision making.

This study established and validated a clinical prediction model, for the occurrence of DCI in patients with aSAH who require mechanical ventilation, with reference to the reporting specifications for the establishment, validation, and updating of clinical prediction models and explored its value in risk prediction to provide a theoretical basis to reduce the incidence of DCI risk in such patients.

## 2. Materials and Methods

### 2.1. Participants and Study Design

The original cohort of this study consisted of patients with aSAH who were treated in a French university hospital's neuro-ICU between 2010 and 2015, with the following inclusion criteria: (I) patients with aSAH hospitalized in the neuro-ICU for mechanical ventilation between January 2010 and December 2015; (II) aged ≥18 years, regardless of sex; (III) subarachnoid hemorrhage due to aneurysm rupture confirmed by computed tomography angiography (CTA); (IV) ST-segment elevation myocardial infarction; and (V) informed consent obtained from the patient or family. The exclusion criteria of the original cohort were as follows: (I) patients with medically induced aneurysm rupture, (II) patients lost to follow-up, and (III) patients with loss of essential clinical features (≥1) after admission and before discharge from the hospital.

In the current study, we performed a post hoc analysis of the dataset shared by Chalard et al. We have added the following exclusion criteria based on the objectives of the study (loss of information is the primary exclusion criterion): missing location of the responsible aneurysm (*n* = 1), missing Glasgow Coma Scale (GCS) scores (*n* = 21), lack of assessment of early brain injury (*n* = 1), lack of mechanical ventilation time (*n* = 2), and missing mRS scores (*n* = 7). After following the above criteria, 204 patients with aSAH who required mechanical ventilation were selected.

Of the 204 patients, 77 were male and 127 were female, and 91 were aged <55 years and 113 were aged ≥55 years; admission World Federation of Neurosurgical Societies (WFNS) grade was III, IV, and V for 19, 81, and 104 patients, respectively, and admission Fisher scores were grades I, II, II, and IV for 1, 4, 37, and 162 patients, respectively.

**Figure 1:**
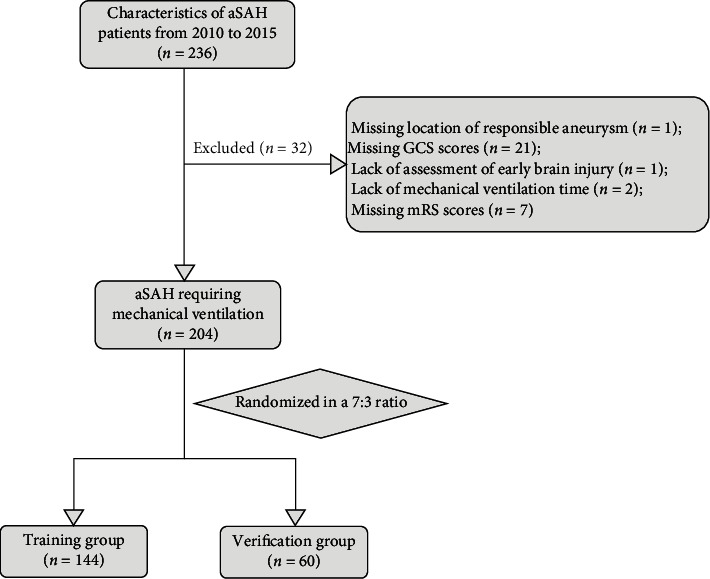
Flowchart of the study selection process. aSAH: aneurysmal subarachnoid hemorrhage.

To develop and validate the clinical prognostic model, 204 patients were randomized using R Studio statistical software in a 7 : 3 ratio to the training group (144 patients) and the verification group (60 patients).  Figure 1 illustrates the flowchart for the selection and randomization of the study population. This study was carried out following the TRIPOD guidelines (see Supplemental File 1 in the Supplementary Material).

### 2.2. Clinical Variables

General data of the patients include sex and age and clinical data include intracerebral hematoma, location of the responsible aneurysm (anterior or posterior circulation), admission GCS score, admission Fisher scores, admission WFNS scores, surgical procedure (craniotomy, endovascular embolization), early brain injury, presence or absence of EVD, angiographic spasm, time to diagnosis and treatment, duration of mechanical ventilation, and prognostic mRS score.

### 2.3. Model Construction and Validation of Queues

The training group was used for univariate analysis, multifactor logistic regression analysis, and construction of nomograms. Factors affecting the occurrence of DCI in patients with aSAH were screened by univariate analysis, and the relationship between each factor and the occurrence of hospital infection in patients was clarified by multifactor logistic regression analysis, and the odds ratio (OR) was used to determine the effect of each factor on the occurrence of hospital infection. The results of the multifactor analysis were visualized and processed through the rms package in the R Studio software to obtain the nomogram. The line segment of the risk value was assigned to the level of value taken by each influencing factor in the model according to the degree of contribution of each influencing factor to the outcome variable. The individual scores were then summed to obtain the total score, and the risk value of the occurrence of the outcome event for that individual was calculated using the functional transformation relationship between the total score and the probability of the occurrence of the outcome event.

The constructed nomograms were examined in the training and the verification groups, where the receiver operating characteristic (ROC) curve was used to verify the model discrimination; the area under the curve (AUC) values were between 0.5 and 1.0, and at AUC > 0.5, the closer the AUC was to 1, the better the diagnosis. When AUC = 0.5, the diagnostic method does not work and has no diagnostic value.

Bootstrap repeated sampling was performed 1000 times to obtain the calibration curve. The Brier value ranged from 0 to 1 and 0 to <0.25, indicating that the model had a predictive value, where the closer the Brier score was to 0, the more accurate the model. The mean absolute error was used to assess consistency between the actual prediction of the model and the corrected prediction, and the closer the mean absolute error was to 0, the better the consistency. Applying the calibration curve with the Hosmer-Lemeshow test (*P* > 0.05 indicates that the results should be accepted, i.e., agreeing that the fitted equation is essentially free of deviation from the true equation, and the higher the *P* value, the better) helped determine the degree of model calibration.

DCA was applied to verify the clinical validity and benefit rate of the model, and the threshold probability indicated the critical probability of patients having DCI after surgery. The benefit rate of the model on the decision curve was higher than that of all patients with DCI who received treatment or of all patients who did not receive DCI and did not receive treatment, indicating the validity and usefulness of the model.

### 2.4. Statistical Analyses

SPSS 26.0 software and R Studio statistical software were used to analyze the data. The Shapiro-Wilk test was used for the normal distribution of continuous variables. The results of normally distributed data are expressed as the mean (standard deviation, SD), and the results of nonparametric data are reported as the median (interquartile range, IQR). Comparisons between groups were made using univariate logistic regression, and differences were considered statistically significant at *P* < 0.05.

## 3. Results

### 3.1. Characteristics of the Participants

In the comparison of information between the training and verification groups, of the 204 patients with aSAH, 144 were in the training group and 60 were in the validation group. None of the differences in clinical information between the training and verification groups were statistically significant (*P* > 0.05) (Table 1).

### 3.2. Risk Factors for DCI

Univariate logistic regression analysis was performed on the training group samples, with statistically significant differences (*P* < 0.05) for EVD, mechanical ventilation, and endovascular coiling. Variables related to *P* < 0.20 were included in the multifactorial logistic regression model using all-inclusive, step-forward, step-backward, and two-way stepwise regression methods. Four models were constructed separately by comparing their AIC values. The two-way regression (model D) was found to be the most appropriate model (the AIC values for each model were attached to model A 162.246, model B 158.453, model C 158.283, and model D 158.283) (Table 2).

Multifactorial logistic regression analysis was used to develop the model. Univariate analysis was used for the initial screening of candidate factors. Candidate factors (*P* < 0.20) in the univariate analysis were entered into a multifactor logistic regression to finalize the independent predictors of DCI risk occurrence to construct a risk prediction scoring system. The occurrence of DCI in patients was included in the multifactor logistic regression analysis as the dependent variable, and the values assigned to each variable are shown in Table 3. The results of the multifactorial logistic regression analysis showed that EVD and rebleeding were independent risk factors for concomitant DCI in patients with aSAH (Table 3).

### 3.3. Construction of Nomograms

The results of the multifactorial analysis were visualized, and a nomogram was constructed, consisting of five indicators: EVD, rebleeding, mechanical ventilation, length of stay, and treatment and their corresponding line segments.

### 3.4. Verification of Nomograms

**Figure 2:**
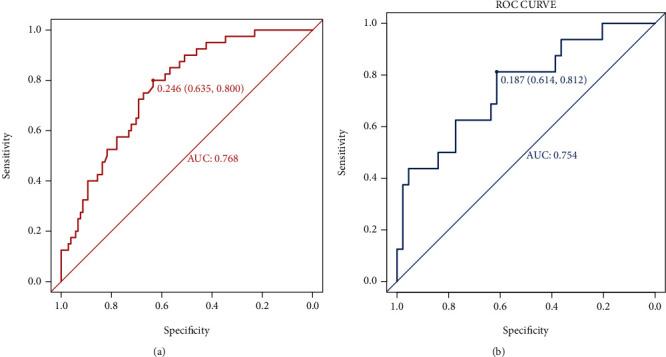
The receiver operating characteristic (ROC) curves of the predictive nomogram for delayed cerebral ischemia (DCI). (a) ROC curve in the training group; the AUC is 0.768 (95% confidence interval: 0.635-0.800); (b) ROC curve in the verification group; the AUC is 0.754 (95% confidence interval: 0.614–0.812).

The constructed model was double-validated in R Studio software for the training and verification groups, and the ROC curve results showed that the model had an AUC of 0.768 (95% confidence interval (CI), 0.635-0.800, *P* < 0.01) in the training group with a threshold probability of 24.6% and an AUC of 0.754 (95% CI, 0.614-0.812) in the verification group with a threshold probability of 18.7% (Figure 2).

**Figure 3:**
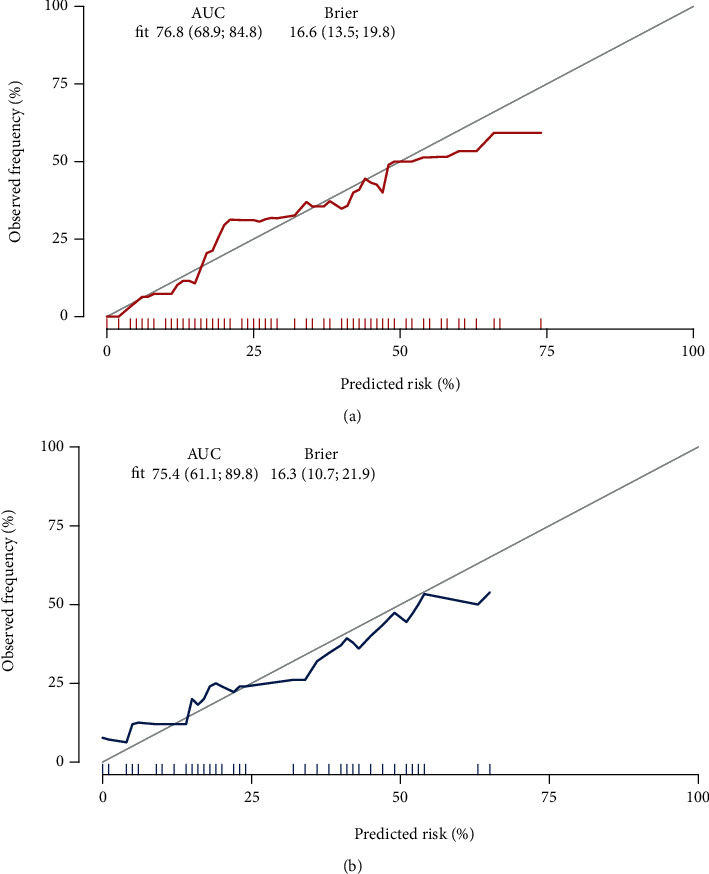
Calibration curves of the predictive nomogram for delayed cerebral ischemia. (a) Calibration curve in the training group; (b) calibration curve in the verification group.

The results of the calibration in the curve analysis of Figure 3 showed that the model had a good agreement between the predicted and actual probabilities of occurrence for the training and verification groups (Brier values of 0.166 and 0.163 points, respectively). Furthermore, the Hosmer-Lemeshow test informed that the *P* values for the training and verification groups were > 0.05 (training group: *x*^2^ = 3.824, and *P* = 0.923; verification group: *x*^2^ = 10.868, *P* = 0.285).

**Figure 4:**
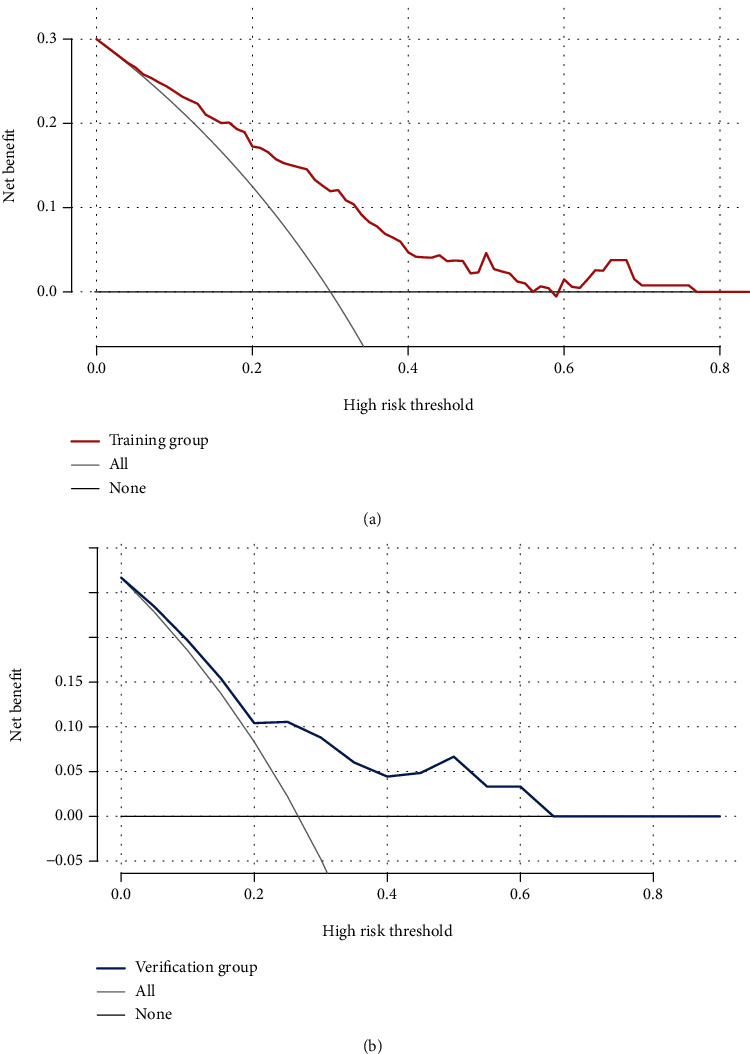
The decision curve analysis (DCA) of the nomogram for DCI. (a) DCA in the training group; (b) DCA in the verification group.

The decision curve analysis (DCA) of the nomograms for the training and verification groups is shown in Figures 3(a) and 3(b), which shows that when using the nomogram to predict the risk of DCI, patients with aSAH may benefit after clinical intervention when the threshold probability for the training and validation groups is 0-77% and 0-63%, respectively (Figure 4).

The threshold probability obtained in the ROC of the test cohort (24.6%) was simultaneously within the threshold probability range of both decision curves in Figure 4; therefore, the model was clinically valid.

**Figure 5:**
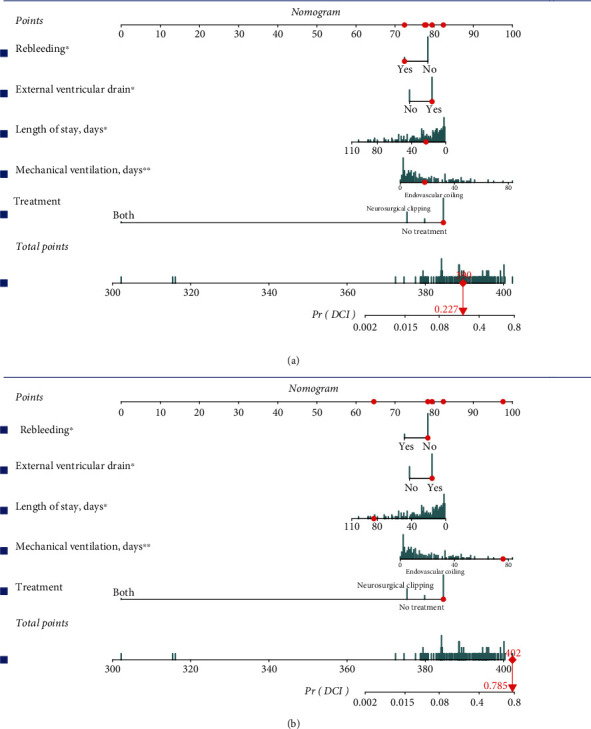
The interactive nomogram. (a) The interactive nomogram of the 4^th^ patient; the probability of developing DCI is 22.7%; (b) the interactive nomogram of the 29^th^ patient; the probability of developing DCI is 78.5%.

As a result of the verification of the above three perspectives (discrimination, calibration, and clinical decision making), we visualized the nomogram using the interactive nomogram. We randomly selected the fourth and twenty-ninth patients in this study and evaluated them using an interactive nomogram. The fourth patient had a lower probability (22.7%) of developing DCI while receiving treatment, while the twenty-ninth patient had a higher probability (78.5%) (Figure 5).

## 4. Discussion

aSAH is a clinical syndrome caused by the rupture of a blood vessel at the base of the skull or on the surface of the brain and the direct flow of blood into the subarachnoid space [21]. A study based on the Chinese population shows that the prevalence of stroke continues to rise in recent years, and hemorrhagic stroke can occur when cerebral vessels are abnormal and become weak or blood pressure is too high [22]. When a hemorrhagic stroke occurs, bleeding can occur in the brain parenchyma (cerebral hemorrhage). aSAH is one of the most dangerous types of SAH and is characterized by treatment difficulty, high disability, and mortality. There are often no obvious clinical symptoms before the rupture of an intracranial aneurysm [23, 24].

Delayed cerebral ischemia is a major factor in the poor prognosis of aneurysmal subarachnoid hemorrhage, and DCI has irreversible and serious consequences once it occurs. Therefore, early prediction and prevention are important [25, 26].

In addition to exploring the specific mechanisms of DCI, various scholars have focused their research on aSAH and attempted to find more precise therapeutic targets and promising therapies to improve the prognosis of patients with aSAH through trends in certain pathophysiological processes or biomarkers [27, 28]. It is of interest that with the development of science and advancement of technology, the use of new biochemical markers of body fluids in the diagnosis and treatment of multiple diseases to predict a patient's disease and the risk of treatment has become the frontier of current technological development. By offering a more accurate prognosis, designing targeted treatment plans, and optimizing the allocation of medical resources, clinicians can predict the likelihood of DCI during the early stages of aSAH. Lin et al. showed that sLOX-1 could effectively serve as a biomarker for DCI after aSAH [29], and Ding et al. found that early neuroglobin levels may play a predictive role in the development of DCI [30]. Furthermore, our previous research confirmed that the expression level of patients with MFG-E8 in the serum of patients with aSAH was associated with the development of DCI [31].

Biological markers, although highly sensitive, require multiple samplings of patients in a staged fashion due to the dynamics of interactions between systems in the body, and the corresponding clinical scores have shown better affordability compared to biological indicators. Different types of prognostic scores have been applied to patients with aSAH, including the GCS score, WFNS, and Fisher score, among others, all of which have been correlated with the severity and prognosis of aSAH [32–34]. However, these scales are unlikely to integrate other risk factors for the patient's comorbidity, leading clinical decision makers to underestimate the patient's prognostic risk and affect the final predictive outcome. Therefore, a more objective predictive model combining multiple factors is necessary, as it allows us to explore the impact of including the evaluation of systems other than the nervous system on the prediction of death and other risks [35–37]. The construction of predictive models to predict the risk of postoperative complications in patients based on the results of multifactorial logistic regression analysis is a current hot topic in many disease studies; however, the results of the logical regression are displayed in the form of formulas, which is time-consuming for users. As an intuitive clinical prediction model, a column line diagram can visualize the results of logistic regression analysis and obtain a graph consisting of each factor and the corresponding line segment, which is easier for the user to understand than a complex formula.

In this study, we used DCI as an entry point to examine risk factors that may be associated with it and included risk factors that may be associated with it outside of the quantitative neurological indicators in the modeling. Few studies have predictive models for aneurysmal subarachnoid hemorrhage, except for predictive risk in clinical fluid samples. In this study, the probability of DCI in this patient was obtained by statistical modeling and quantitative assessment of each risk factor in the model using a simple and easy-to-use column line graph. This model can help clinicians develop the most beneficial treatment plan for patients in early decision making, consistent with the principle of individualized treatment.

Our study ultimately incorporated five factors (EVD, rebleeding, duration of mechanical ventilation, days of hospitalization, and treatment modality) to construct the final predictive column line graph and to elucidate the validity of the model from three perspectives: mechanism, theoretical basis, and statistical validation.

An EVD can be combined with postoperative monitoring results to promptly adjust drainage to ensure the drainage effect and achieve an effective reduction in intracranial pressure. It can slowly release bloody cerebrospinal fluid and reduce stimulation damage to cerebral vessels and brain tissue, improve blood supply to brain tissue, reduce ischemic and hypoxic damage to brain tissue, facilitate inhibition of progressive progression of neurological deficits, and improve prognosis [38, 39].

To our surprise, the risk score was higher for patients who underwent external ventricular drain (EVD) (15 points) than for those who did not (0 points), and we reasoned that the prevention of DCI depends on maintaining adequate cerebrovascular volume and that EVD plays a negative regulatory role in maintaining blood volume, which is worthy of a follow-up study.

Rebleeding and DCI after aSAH have been reported to be highly morbid and potentially fatal events that appear to have an independent negative impact on overall functional outcomes. However, early rebleeding did not significantly affect the risk of delayed ischemic complications, and rebleeding after aSAH was associated with multiple somatic and neurological complications, leading to increased morbidity and mortality, but not with changes in the incidence or timing of DCI [40, 41]. The above findings are consistent with our modeling results: the risk assignment for patients who experienced rebleeding was 0.

As patients receive mechanical ventilation for longer periods, the risk of nosocomial infection increases significantly; therefore, the DCI risk score increases in steps [42–44]. However, why is the risk lower with a longer hospital stay? Is it related to specialized medical conditions and timely testing in hospitals? If the number of days in the hospital and the duration of mechanical ventilation result in opposite risk outcomes, we should consider how to develop a more precise treatment plan for the right number of days in the hospital and whether measures can be taken to get patients off the ventilator as early as possible for long hospital stays.

Endovascular coiling for the treatment of cerebral aneurysms can achieve better clinical outcomes than open clipping and has obvious minimally invasive advantages. However, the current cost of vascular intervention is relatively high and may be the procedure of choice for this disease when economic conditions allow it [45, 46]. However, for patients with high-grade aSAH who require intensive care in this study, the outcome of endovascular coiling has been shown to be not superior to clipping in patients with high-grade aSAH, and embolization carries a higher risk of death [47].

In addition to previous studies and the theoretical basis, the risk model developed in this study, from a statistical perspective, is notable in terms of differentiation, calibration, and clinical decision making, and the model has both theoretical and practical values in providing individualized treatment options for patients with aSAH who require mechanical ventilation.

The limitations of this study are as follows. The included patient population is a single-center group, which is not well represented, and the sample size and case sources are expected to be further expanded in the future. In addition, the study did not include information on patients' tests, such as routine blood chemistry, and it would have been better to include quantitative indicators of the course of specific changes in vital signs. However, the results of this study provide a clear direction for a reasonable prediction of the risk of serious complications, such as DCI in patients requiring intensive care and mechanical ventilation, which would help clinicians better identify the population at risk and help them make appropriate clinical decisions. For example, if a patient is determined to be at higher risk of developing DCI as per nomogram, early investigation measures, such as transcranial Doppler ultrasound or cerebral perfusion imaging to determine if a patient has developed cerebral vasospasm, and the use of nimodipine to prevent and maintain normal cerebrovascular volumes are essential, while in patients at low risk, overmedication can be avoided.

## 5. Conclusions

Based on logistic regression analysis, the predictive model for concurrent DCI in aSAH can theoretically and practically be validated and provide individualized treatment alternatives for patients with aSAH who require mechanical ventilation.

## Figures and Tables

**Table 1:** Comparison of the demographic and clinical data between training and verification groups.

Age (years) (%)	Train (*n* = 144)	Validation (*n* = 60)	*P* value
<55	65 (45.14)	26 (43.33)	0.9348
≥55	79 (54.86)	34 (56.67)	
Gender (%)			
Male	58 (40.28)	19 (31.67)	0.3185
Female	86 (59.72)	41 (68.33)	
Modified Rankin scale (mRS) (%)			
No	40 (27.78)	23 (38.33)	0.1866
Yes	104 (72.22)	37 (61.67)	
Aneurysm localization (%)			
Anterior	118 (81.94)	52 (86.67)	0.5363
Posterior	26 (18.06)	8 (13.33)	
Intracerebral hemorrhage (%)			
No	80 (55.56)	26 (43.33)	0.1504
Yes	64 (44.44)	34 (56.67)	
Early brain injury (%)			
No	38 (26.39)	19 (31.67)	0.5523
Yes	106 (73.61)	41 (68.33)	
External ventricular drain (EVD) (%)			
No	40 (27.78)	26 (43.33)	0.0455
Yes	104 (72.22)	34 (56.67)	
Rebleeding (%)			
No	122 (84.72)	55 (91.67)	0.2683
Yes	22 (15.28)	5 (8.33)	
Angiographic vasospasm (%)			
No	93 (64.58)	41 (68.33)	0.7247
Yes	51 (35.42)	19 (31.67)	
Hypertension (%)			
Normal	97 (67.36)	36 (60.00)	0.4662
I	12 (8.33)	8 (13.33)	
II	35 (24.31)	16 (26.67)	
GCS (%)			
≤5	58 (40.28)	26 (43.33)	0.9064
6~8	49 (34.03)	20 (33.33)	
≥9	37 (25.69)	14 (23.33)	
WFNS (%)			
III	12 (8.33)	7 (11.67)	0.7009
IV	59 (40.97)	22 (36.67)	
V	73 (50.69)	31 (51.67)	
Fisher (%)			
I	1 (0.69)	0 (0.00)	0.72
II	2 (1.39)	2 (3.33)	
III	27 (18.75)	10 (16.67)	
IV	114 (79.17)	48 (80.00)	
Delay between diagnosis and treatment (%)			
<12 h	84 (58.33)	35 (58.33)	0.4449
12~24 h	39 (27.08)	14 (23.33)	
24~48 h	3 (2.08)	4 (6.67)	
>48 h	3 (2.08)	3 (5.00)	
Failure	5 (3.47)	1 (1.67)	
Withdrawal of care	10 (6.94)	3 (5.00)	
Treatment (%)			
No treatment	15 (10.42)	4 (6.67)	0.5415
Endovascular coiling	92 (63.89)	35 (58.33)	
Neurosurgical clipping	35 (24.31)	20 (33.33)	
Coiling and clipping	2 (1.39)	1 (1.67)	
Delayed cerebral ischemia, DCI (%)			
No	104 (72.22)	44 (73.33)	1
Yes	40 (27.78)	16 (26.67)	
Mechanical ventilation (days) (median [IQR])	12.000 [5.000, 27.250]	11.000 [6.000, 20.500]	0.6326
Length of stay (days) (median [IQR])	18.500 [7.000, 38.000]	17.000 [7.750, 31.000]	0.58

**Table 2:** Univariate logistic regression analysis of DCI in patients in training group.

Characteristics	*β*	SE	OR	95% CI	*Z*	*P* value
Age	-0.41	0.374	0.66	0.32-1.38	-1.098	0.272
Gender	0.016	0.38	1.02	0.48-2.14	0.042	0.966
Modified Rankin scale (mRS)	0.575	0.449	1.78	0.74-4.29	1.282	0.2
Aneurysm localization	-0.572	0.537	0.56	0.2-1.62	-1.065	0.287
Intracerebral hemorrhage	-0.692	0.39	0.5	0.23-1.07	-1.773	0.076
Early brain injury	-0.748	0.403	0.47	0.21-1.04	-1.856	0.063
External ventricular drain (EVD)	1.561	0.566	4.76	1.57-14.45	2.759	0.006
Rebleeding	-1.014	0.652	0.36	0.1-1.3	-1.556	0.12
Angiographic vasospasm	21.857	1838.554	3107401561	0-Inf	0.012	0.991
Mechanical ventilation	0.024	0.011	1.02	1-1.05	2.25	0.024
Length of stay	0.012	0.008	1.01	1-1.03	1.561	0.119
Hypertension I	-0.146	0.704	0.86	0.22-3.43	-0.207	0.836
Hypertension II	0.036	0.437	1.04	0.44-2.44	0.083	0.934
GCS 6~8	-0.073	0.448	0.93	0.39-2.24	-0.163	0.871
GCS ≥ 9	0.44	0.457	1.55	0.63-3.8	0.964	0.335
WFNS IV	0.431	0.721	1.54	0.37-6.32	0.597	0.55
WFNS V	-0.094	0.722	0.91	0.22-3.75	-0.13	0.897
Fisher II	-33.132	2938.83	0	0-Inf	-0.011	0.991
Fisher III	-17.097	2399.545	0	0-Inf	-0.007	0.994
Fisher IV	-17.641	2399.545	0	0-Inf	-0.007	0.994
Delay between diagnosis and treatment 12~24 h	0.166	0.415	1.18	0.52-2.66	0.399	0.69
Delay between diagnosis and treatment 24~48 h	0.166	1.248	1.18	0.1-13.62	0.133	0.894
Delay between diagnosis and treatment > 48 h	-16.707	2284.102	0	0-Inf	-0.007	0.994
Delay between diagnosis and treatment failure	-0.528	1.143	0.59	0.06-5.54	-0.462	0.644
Delay between diagnosis and treatment withdrawal of care	-16.707	1251.054	0	0-Inf	-0.013	0.989
Treatment endovascular coiling	2.105	1.057	8.21	1.03-65.15	1.991	0.047
Treatment neurosurgical clipping	0.847	1.142	2.33	0.25-21.88	0.742	0.458
Treatment coiling and clipping	-12.927	1029.122	0	0-Inf	-0.013	0.99

**Table 3:** Multivariate logistic regression analysis of DCI after aSAH requiring mechanical ventilation.

Characteristics	*β*	SE	OR	95% CI	*Z*	*P* value
External ventricular drain (EVD)	1.379	0.616	3.97	1.19-13.28	2.239	0.025
Rebleeding	-1.525	0.691	0.22	0.06-0.84	-2.208	0.027
Mechanical ventilation	0.058	0.031	1.06	1-1.13	1.885	0.059
Length of stay	-0.037	0.024	0.96	0.92-1.01	-1.536	0.125
Treatment endovascular coiling	1.322	1.125	3.75	0.41-34.03	1.175	0.24
Treatment neurosurgical clipping	-0.03	1.219	0.97	0.09-10.59	-0.024	0.981
Treatment coiling and clipping	-14.5	1407.446	0	0-Inf	-0.01	0.992

## Data Availability

The datasets generated and/or analyzed during the current study are available in the Dryad repository “doi:10.5061/dryad.47d7wm3b4.”

## Supplementary Materials

Supplementary Materials Supplemental Files 1: the TRIPOD guidelines.
